# A successful case of anti-NMDAR encephalitis without tumor treated with a prolonged regimen of plasmapheresis

**DOI:** 10.1590/S1980-57642014DN81000014

**Published:** 2014

**Authors:** Mateus Mistieri Simabukuro, Rafael Gustavo Sato Watanabe, Lécio Figueira Pinto, Carla Guariglia, Daniella Costa de Menezes e Gonçalves, Renato Anghinah

**Affiliations:** 1 Neurology Department, Hospital Samaritano, São Paulo, Brazil.; 2 Neurology Department, Hospital das Clínicas of the University of São Paulo School of Medicine (HCFMUSP), São Paulo, Brazil.; 3 Infectology Department, Hospital Samaritano, São Paulo, Brazil.

**Keywords:** autoimmune disease, encephalitis, anti-NMDA receptor, plasmapheresis, psychiatric symptoms

## Abstract

Anti-NMDA receptor encephalitis is a severe but treatable autoimmune disease of the CNS. However, the use of immunotherapy and long-term outcomes have yet to be defined for this disease. We describe a case of an 18-year-old male diagnosed with anti-NMDAR encephalitis not associated with tumor, which did not respond to initial treatment with immunoglobulin, followed by corticosteroids, cyclophosphamide and evolved with significant clinical improvement after a prolonged course of plasmapheresis. Although it is not possible to affirm the good outcome was due solely to the prolonged plasmapheresis regimen, recently published data shows that improvement may take weeks or months to occur. This case discloses another therapeutic possibility for patients with refractory disease who fail to respond to recommended first-line and second-line therapy.

## Abstract (pt)

A encefalite antirreceptor de NMDA é uma grave doença autoimune do sistema nervoso central, mas responsiva a tratamento. No entanto o uso da imunoterapia e o prognóstico a longo prazo ainda não foram estabelecidos para esta doença. Aqui nós descrevemos o caso de um homem de 18 anos de idade diagnosticado com encefalite antirreceptor de NMDA, não associada a tumor, o qual provavelmente não respondeu à terapia inicial com corticosteroides, ciclofosfamida e, o qual evoluiu com melhora clínica importante após um curso prolongado de plasmaférese. Embora não seja possível atribuir o desfecho favorável apenas ao regime de plasmaférese prolongado, uma vez que publicações recentes demonstram que a resposta à imunoterapia pode levar semanas à meses, nosso caso abre uma possibilidade terapêutica nova para casos com doença refratária, não responsivos ao tratamento de primeira e segunda-linha preconizados

## CASE PRESENTATION

An 18-year-old male presented with numbness to the left side of his face and insomnia. In the days that followed, behavioral and psychiatric symptoms consisting of agitation, irritability and delusional thoughts manifested. He was initially assessed by a neurologist, who prescribed clonazepam and sertraline. Because psychomotor agitation worsened, treatment was changed to valproate, risperidone and promethazine. After eleven days, focal motor seizures developed. On day 15, he presented with a decreased level of consciousness, evolved with urinary incontinence and hyperthermia. He was admitted to another hospital on the 17^th^ day. In the initial investigation workup, cerebrospinal fluid (CSF) revealed mild lymphocytic pleocytosis (25 mm^3^), normal protein concentration (39 mg per dL), normal glucose concentration (59 mg per dL), immunologic reactions for syphilis and toxoplasmosis were negative, polymerase chain reaction for Varicella Zoster, Herpes simplex virus 1 and 2 tested negative, and IgG was slightly elevated (12.8%). Brain magnetic resonance imaging (MRI) showed unremarkable findings. Electroencephalogram (EEG) showed disorganization of background activity and slow wave paroxysms. Acyclovir was then initiated. Hyperthermia persisted, so malignant neuroleptic syndrome was then suspected and he was given dantrolene. On day 19 he was submitted to endotracheal intubation for barbiturate-induced coma to treat refractory epileptic status. On day 25, he was transferred to our hospital. The patient was empirically started on doxycycline, sulphamethoxazole-trimethoprim, ampicillin and dexamethasone. Anticonvulsants were adjusted. Investigation workup was broadened to include autoimmune and paraneoplastic causes of encephalitis. Blood samples for anti-NMDA receptor were collected and sent for analysis (CSF samples were not sent). Chest, abdominal and pelvic computed tomography (CT) scans showed an enlarged axillary lymph node. Biopsy was performed but pathologic findings suggested reactive hyperplasia. The paraneoplastic panel was negative (only serum sample was analysed) for anti-NMDA; Anti-Neuronal Nuclear Antibody 1 (ANNA1), ANNA-2, ANNA-3; Anti-Glial/Neuronal Nuclear Ab Type 1 (AGNA-1); Purkinje cell cytoplasmic antibody type 1 (PCA-1), PCA-2, PCA-Tr; Amphy-sine antibodies; Collapsin response mediator protein 5 (CRMP-5); anti-voltage-gated calcium channel (VGCC); antivoltage-gated potassium channel (anti-VGKC); anti-myelin-associated glycoprotein (anti-MAG); Ganglionic acetylcholine receptor autoantibody and acetylcholine autoantibodies. Borrelia Burgdorferi enzyme-linked immunosorbent assay (ELISA) tested positive, but Western blot test was negative.

Because clinical features were highly suggestive of anti-NMDA receptor encephalitis, a diagnostic test was repeated, this time including CSF samples. Results disclosed antibodies against anti-NMDA receptor detected only in the CSF.

Intravenous and continuous midazolam, propofol, and ketamine were started to wean patient from barbituric burst-suppression pattern on EEG. On day 29, intravenous immunoglobulin (0.4 mg/Kg/day for five days) was started empirically. In the ensuing days, there was a slight improvement in the neurological conditions; the patient was submitted to tracheostomy. Antiepileptic drugs were changed, midazolam, propofol and ketamine were weaned, but the patient was still comatose and although non-convulsive status epilepticus was resolved, he still had sporadic seizures. On day 36, the patient was still comatose and presenting autonomic instability, when methylprednisolone was initiated. On day 39, he received cyclophosphamide. The patient began to present spontaneous eye opening and followed the examiner, although he did not obey verbal commands and showed no response to painful stimuli. On day 42, he began obeying verbal commands, showed a slight improvement in neurological status and no seizures, but oro-lingual-facial dyskinesias were still present. On day 45, his level of consciousness deteriorated again. Hence, the patient was submitted to the first plasmapheresis session. After the third plasmapheresis session, on day 48, the patient was able to talk (assisted by a phonation valve) and exhibited a significant improvement in level of consciousness. A regular plasmapheresis regimen (at least two times a week) was maintained and the patient gradually recovered: antiepileptic drugs doses were reduced, hyperthermia and tachycardia resolved and decannulation was achieved.

After marked recovery, he was discharged, twelve days after the 19^th^ plasmapheresis session. At discharge, his mental examination was normal and neurologic examination showed bilateral foot drop and absent ankle reflex, where these findings were considered to be due to critical care neuropathy.

## DISCUSSION

In 2005, a syndrome affecting young women with ovarian teratoma characterized by prominent psychiatric symptoms, memory dysfunction, decreased level of consciousness and central hypoventilation was described.^1^ Shortly after, the discovery of a specific autoantibody to N-methyl D-aspartate receptor (NMDAR) in serum and CSF of these 4 patients, and of another 8 patients with similar neurologic symptoms (7 with associated ovarian teratoma), changed the paradigm in CNS autoimmunity^2^ neuropathological findings, tumors, and serum/cerebrospinal fluid antibodies using rat tissue, neuronal cultures, and HEK293 cells expressing subunits of the N-methyl-D-aspartate receptor (NMDAR).

It is believed to be the second-most-common cause of autoimmune encephalitis after acute demyelinating encephalomyelitis (ADEM).^3^

Care must be taken in performing diagnostic tests and antibody studies should be done in both serum and CSF because NMDAR antibodies might go undetected in serum after immunotherapy initiation or delayed diagnosis^4^ the encephalitis associated with antibodies against the N-methyl-D-aspartate receptor (NMDAR). In our case antibodies were detected only in CSF.

Although severe, this form of autoimmune encephalitis is potentially responsive to treatment, which centers on two principles: immunotherapy (first and second line) and tumor removal, if applicable. A recent observational worldwide cohort study, in which 577 patients were enrolled, demonstrated that the occurrence of an underlying tumor was greater in female patients aged 12 years or older than in young children and male patients, and if present is almost always an ovarian teratoma containing nervous tissue and expressing NMDAR (94% of all tumors were ovarian teratomas).^5^ First-line therapy consists of methylprednisolone plus IVIg or plasmapheresis while second-line therapy is rituximab, cyclophosphamide or both.

In the study, 472 patients were treated with first-line immunotherapy (steroids, intravenous immunoglobulin, plasmapheresis) or tumor removal, 253 patients (53%) showed symptom improvement within 4 weeks of treatment; 125 patients (57%) received second-line immunotherapy (rituximab or cyclophosphamide), and of these 84 had an mRS score of 0-2 during the first 24 months.

Plasmapheresis is a first-line therapy option^4,5^ the encephalitis associated with antibodies against the N-methyl- D-aspartate receptor (NMDAR, however evidence for its efficacy is very limited, mostly derived from case reports and expert opinion.^6-9^ It has been used for other paraneoplastic neurological syndromes (subacute cerebellar degeneration, paraneoplastic encephalomyelitis, paraneoplastic opsoclonus/myoclonus, and cancer-associated retinopathy). The findings of specific autoantibodies in these syndromes led to the use of plasmapheresis in their management.^10^ For these syndromes, the American Society for Apheresis assigns a category III (grade 2 C) recommendation.^11^ However, effects of individual treatments (e.g. Plasma exchange vs. IVIg) cannot be compared side-by-side statically.^5^

Although it is difficult to establish an unequivocal causal relationship between plasmapheresis and improvement in this case because other modalities of immunotherapy were used beforehand, our patient showed a significant clinical improvement concomitant with plasmapheresis initiation.
